# Near-Infrared Spectroscopy Usefulness in Validation of Hyperventilation Test

**DOI:** 10.3390/medicina58101396

**Published:** 2022-10-06

**Authors:** Stefan Sandru, Dan Buzescu, Carmen Denise Mihaela Zahiu, Ana Spataru, Anca Maria Panaitescu, Sebastian Isac, Cosmin Ion Balan, Ana-Maria Zagrean, Bogdan Pavel

**Affiliations:** 1 Department of Functional Sciences, Carol Davila University of Medicine and Pharmacy, 050474 Bucharest, Romania; 2 Department of Critical Care, King’s College Hospital Denmark Hill, London SE5 9RS, UK; 3 Department of Obstetrics and Gynecology, Filantropia Clinical Hospital Bucharest, 011171 Bucharest, Romania; 4 Department of Obstetrics and Gynecology, Carol Davila University of Medicine and Pharmacy, 050474 Bucharest, Romania; 5 Department I of Cardiovascular Anesthesiology and Intensive Care, “Prof. C. C Iliescu” Emergency Institute for Cardiovascular Diseases, 050474 Bucharest, Romania

**Keywords:** NIRS, hyperventilation, hypocapnia, cerebral blood flow

## Abstract

*Background*: The hyperventilation test is used in clinical practice for diagnosis and therapeutic purposes; however, in the absence of a standardized protocol, the procedure varies significantly, predisposing tested subjects to risks such as cerebral hypoxia and ischemia. Near-infrared spectroscopy (NIRS), a noninvasive technique performed for cerebral oximetry monitoring, was used in the present study to identify the minimum decrease in the end-tidal CO_2_ (ETCO_2_) during hyperventilation necessary to induce changes on NIRS. *Materials and Methods*: We recruited 46 volunteers with no preexisting medical conditions. Each subject was asked to breathe at a baseline rate (8–14 breaths/min) for 2 min and then to hyperventilate at a double respiratory rate for the next 4 min. The parameters recorded during the procedure were the regional cerebral oxyhemoglobin and deoxyhemoglobin concentrations via NIRS, ETCO_2_, and the respiratory rate. *Results*: During hyperventilation, ETCO_2_ values dropped (31.4 ± 12.2%) vs. baseline in all subjects. Changes in cerebral oximetry were observed only in those subjects (*n* = 30) who registered a decrease (%) in ETCO_2_ of 37.58 ± 10.34%, but not in the subjects (*n* = 16) for which the decrease in ETCO_2_ was 20.31 ± 5.6%. According to AUC-ROC analysis, a cutoff value of ETCO_2_ decrease >26% was found to predict changes in oximetry (AUC-ROC = 0.93, *p* < 0.0001). Seven subjects reported symptoms, such as dizziness, vertigo, and numbness, throughout the procedure. *Conclusions*: The rise in the respiratory rate alone cannot effectively predict the occurrence of a cerebral vasoconstrictor response induced by hyperventilation, and synchronous ETCO_2_ and cerebral oximetry monitoring could be used to validate this clinical test. NIRS seems to be a useful tool in predicting vasoconstriction following hyperventilation.

## 1. Introduction

The hyperventilation procedure was first described 50 years ago by Major Meyer Friedman with the intent of providing a diagnostic tool for approaching patients with dyspnea [1]. While initially designed to differentiate between organic and psychosomatic etiologies of dyspnea, currently, the hyperventilation test is widely used in clinical practice for the evaluation of vestibular abnormalities or for the assessment of the vascular reactivity. From a physiological point of view, the changes that occur during hyperventilation are mediated by the reduction in the partial pressure of arterial carbon dioxide (PaCO_2_), which, in turn, interferes with the cerebrovascular autoregulatory functions and leads to vasoconstriction [2]. Vasoconstriction is accompanied by a lower regional blood flow, which results in a reduced oxygen supply and an increased risk of ischemic phenomena [3,4]. 

At present, there are few indications for the use of the hyperventilation procedure in clinical practice, whether by voluntary control of the breathing or by using mechanical ventilation devices. Some of these are the diagnosis and the study of epileptic seizures, the reduction in elevated intracranial pressure in neurosurgery (e.g., for the control of post-traumatic intracranial hypertension), and the coronary artery vasospasm test in patients with suspected or documented vasospastic angina [5,6,7]. A special indication of the use of the hyperventilation test is the training of pilots in aero-spatial medicine. Flight pilots are prone to hyperventilation in emergencies or during rapid decompressions of the cabin; accordingly, they need to have a good tolerance to hyperventilation [8]. Thus, they should pass a correctly performed hyperventilation test to avoid seizures or angina during the hypocapnia-induced cerebral vasoconstriction.

Classically, during a hyperventilation test, subjects are asked to hyperventilate vigorously for a few minutes at a respiratory rate that is higher than normal [9]. A physiological response in the form of angina, tinnitus, or dizziness is recorded thereafter. To date, there have been limited attempts to standardize the test in terms of ventilatory rate or duration. A quick survey of the current medical literature identified significant variations in the procedure’s methodology from study to study, both in terms of test duration, with ranges from 3 and 5 min up to 20 and 60 min, and in terms of the prescribed respiratory frequency [10,11]. It is also well known that humans have large variability in terms of respiratory depth and rate during hyperventilation, which limits the applicability of standardization based on clinical criteria only [12]. 

The purpose of the present study was to evaluate objectively and in a noninvasive manner the changes that occur in the cerebral blood flow during the hyperventilation procedure. We used end-tidal CO_2_ in non-intubated patients to quantify the extent of hypocapnia in subjects whose cerebral perfusion was estimated using near-infrared spectroscopy (NIRS) technology. NIRS technology noninvasively measures the concentration of oxygenated and deoxygenated hemoglobin, as well as derived parameters, in the target tissues, offering information about the perfusion and the oxygen supply of the tissue [13,14]. NIRS is used in clinical practice for the assessment of the cerebral oximetry during various states as traumatic brain injury, carotid endarterectomy, stroke, epilepsy, cardiac surgery, migraine, and mild cognitive impairment, and it has the advantage of providing real-time values of oxygenated an deoxygenated hemoglobin, in addition to being a noninvasive technique with no contraindications [15,16]. Another advantage of this technique is represented by the high reproducibility of the experiments. As a result, we assessed the degree of hypocapnia required to induce changes in the cerebral blood flow. NIRS was previously used successfully to assess the cerebral autoregulation [17]. Additionally, we assessed the objective changes at different timepoints to establish the minimal duration of hypocapnia compatible with an accurate hyperventilation test. 

## 2. Materials and Methods

For this study, 46 healthy volunteers, 31 men and 15 women, aged between 18 and 38 years old (with a median age of 22 years) have been recruited. Exclusion criteria for participating in the study were as follows: history of epilepsy, seizure(s), stroke, vertigo, anxiety, myocardial infarction, cardiac arrhythmias, congenital heart diseases, asthma, severe pulmonary fibrosis, chronic obstructive pulmonary disease, tuberculosis, current upper respiratory tract infection, and peripheral artery disease. Prior to being enrolled in the study, each volunteer was informed regarding the methodology of the procedure. An anamnestic evaluation of the candidates was carried out to identify and exclude potential subjects with medical conditions that could suffer decompensation in the context of hyperventilation. Afterward, the selected candidates considered appropriate for the test signed an informed consent form. The procedure was conducted in baseline conditions, at rest, in the morning, with no prior consumption of tobacco, caffeine, or other medications for the previous 12 h.

The parameters monitored during the hyperventilation procedure were (1) the variation of oxyhemoglobin and deoxyhemoglobin using near-infrared spectroscopy technology (NIRS) (PortaLite, Artinis Medical Systems, Einsteinweg, The Netherlands), which is an indirect parameter for estimating cerebral perfusion [18], and (2) the partial pressure of carbon dioxide in the expired air (ETCO_2_) and the respiratory rate (RR) using a Vamos gas monitor (Dräger, Germany) [19]. NIRS-driven oximetry allowed the monitoring of cerebral blood oxyhemoglobin and deoxyhemoglobin concentrations by placing a NIRS oximeter sensor on the subject’s forehead. Preparing for the procedure, the NIRS oximeter sensor was applied to the subject’s forehead (on the right side), and they were offered a facial mask connected to the capnograph device through which they breathed, thus allowing for full data acquisition. If any artefact motion was identified on the NIRS trace, we asked to subjects to repeat the test after 1 h. Fortunately, with many of them being medical students, we had to repeat the test in just two cases.

Before beginning to hyperventilate, the subject was instructed to breathe with a respiratory rate in a relaxed state for 2 min, after which each subject was instructed to double their respiratory rate (following the respiratory rate displayed by the capnograph) for the next 4 min. The values for each of the monitored parameters were registered at 2 min with respect to 4 min after hyperventilation started. Oximetry analysis was performed continuously over the course of the procedure, preceding the initiation of hyperventilation, and during hyperventilation. At the end of the 4 min of hyperventilating, the subjects were instructed to return to their basal respiratory rates, while oximetry monitoring continued to objectivize post-hyperventilation oximetry changes. We considered that a patient was responsive to hyperventilation if the oxyhemoglobin decreased during hyperventilation by more than 50% of its basal value. 

Statistical analysis. Our data are presented as the average values ± standard deviation. Student’s t-test was performed for mean comparison and determination of area under the receiver operator’s characteristics curve (AUC-ROC). AUC-ROC analysis was used to evaluate the performance of ETCO_2_ in predicting NIRS change during hyperventilation. The results were considered statistically significant for *p* < 0.05. NCSS 2022 Statistical Software (NCSS 2022, LLC, Kaysville, UT, USA) was used for data analysis. All patients provided written informed consent, and the study was approved by the Clinical Emergency Hospital for Plastic, Reconstructive, and Burns Surgery Ethics Committee (No. 2/19.06.2020).

## 3. Results

Analyzing the data acquired during the procedure, we observed that the partial pressure of carbon dioxide in the expired air (measured through capnography as ETCO_2_) diminished in all subjects as a result of hyperventilation (%ETCO_2_ decreased on average by 31.4 ± 12.2%). The values of the respiratory rate and end-tidal CO_2_ are presented in Table 1. There was no statistically significant difference between the value of ETCO_2_ measured at 2 min and the value of ETCO_2_ measured at 4 min (25.89 ± 4.95% and 24.6 ± 5.85%, respectively; *p* = 0.24). This observation is an argument in favor of limiting the duration of the hyperventilation procedure to 4 min. 

**Figure 1:**
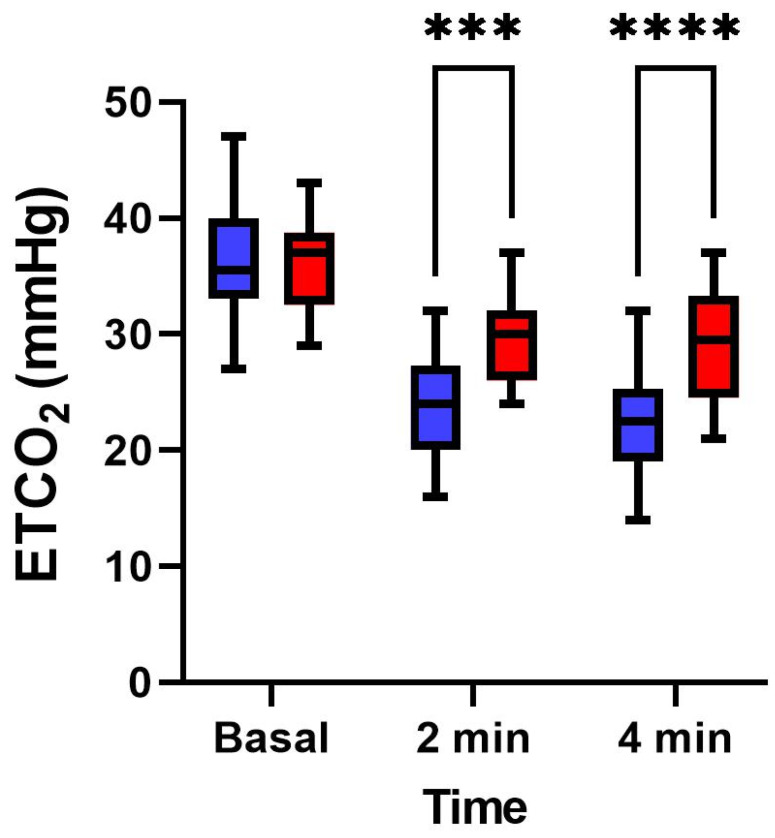
Mean values of end-tidal CO_2_ (ETCO_2_) in group presenting changes in the cerebral oximetry (blue) and in group not presenting any change in cerebral oximetry (red) at baseline and during hyperventilation at 2 min and 4 min. *** *p* < 0.001; **** *p* < 0.0001.

In Figure 1, we can observe that, during basal ventilation, there anywhere no differences between the groups (35.96 ± 4.72 vs. 36.18 ± 4.16; *p* = 0.87) in end-tidal CO_2_, whereas, at 2 min (23.86 ± 4.2 vs. 29.68 ± 4.01) and 4 min (22.4 ± 4.58 vs. 28.75 ± 4.97), there were significant differences between the group which presented changes in oximetry (blue bars) and group which did not (red bars) (*p* < 0.01).

Seven subjects of the 46 (15%) described the occurrence of symptoms such as dizziness, vertigo, and sensations of paresthesia in the extremities. 

**Figure 2:**
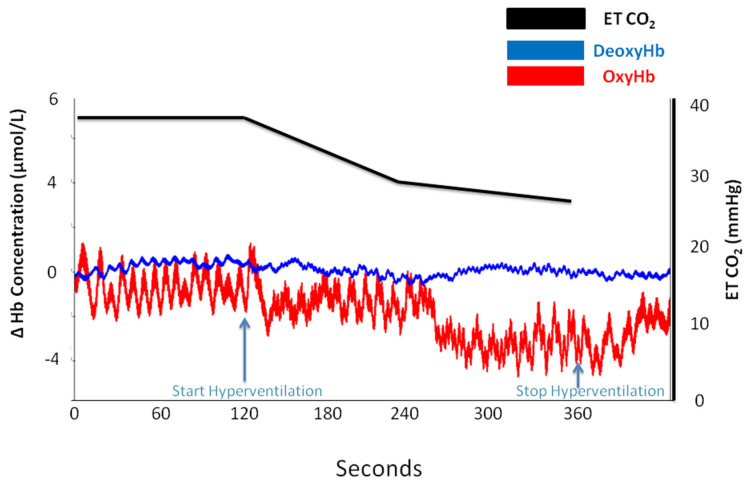
Oxyhemoglobin (oxyHb) and deoxyhemoglobin (deoxyHb) and end-tidal CO_2_ changes at baseline and during hyperventilation and post-hyperventilation periods in the case of a subject who presented a drop in oxyhemoglobin.

**Figure 3:**
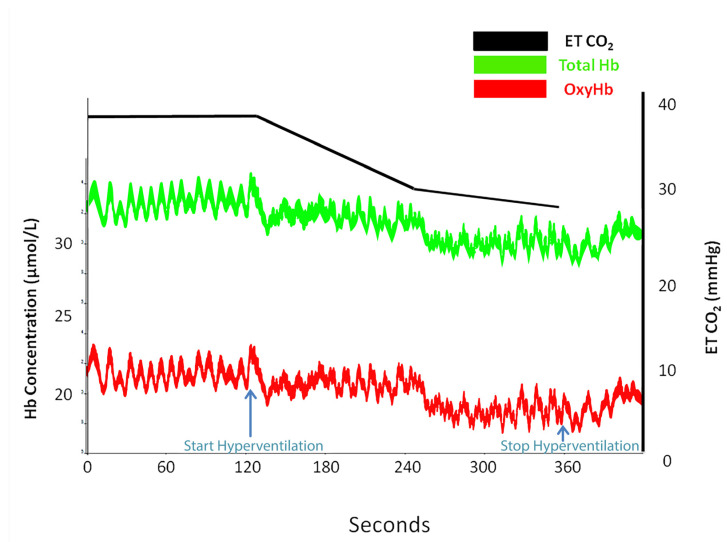
Total hemoglobin (tHb) trace (green), oxyhemoglobin trace (red), and end-tidal CO_2_ trace (black) at baseline and during hyperventilation in the subject presented in Figure 2.

**Figure 4:**
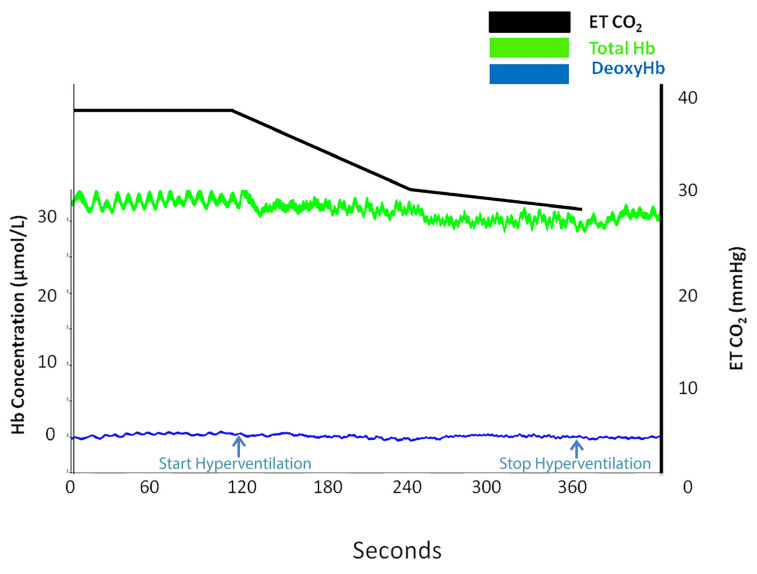
Total hemoglobin (tHb) trace (green), deoxyhemoglobin trace (blue), and end-tidal CO_2_ trace (black) at baseline and during hyperventilation in the subject presented in Figure 2.

**Figure 5:**
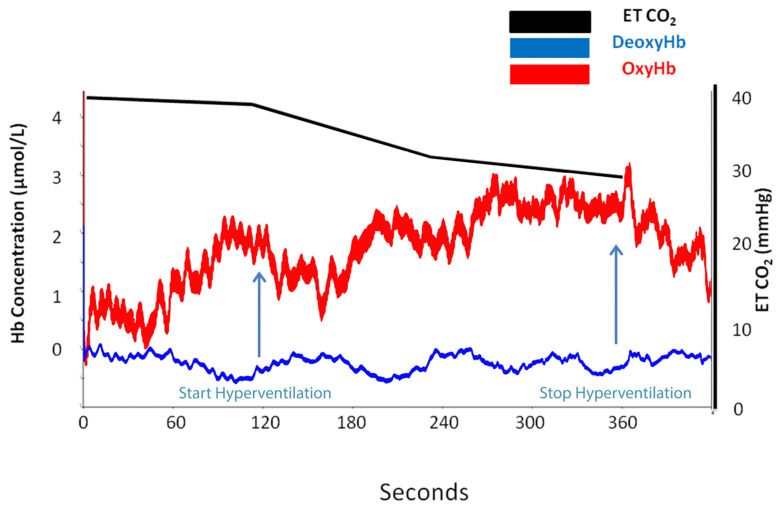
Oxyhemoglobin (oxyHb), deoxyhemoglobin (deoxyHb), and end-tidal CO_2_ changes at baseline and during hyperventilation and post-hyperventilation periods in the case of a subject who did not present any drop in oxyhemoglobin.

However, oximetry changes were seen only in those subjects (*n* = 30) who registered a reduction in ETCO_2_ (%) of 37.58 ± 10.34 compared with 20.31 ± 5.6 in subjects (*n* = 16) who did not present any signs on NIRS (Table A2). Figure 2, Figure 3, Figure 4 and Figure 5 are charts representing the oximetry and end-tidal CO_2_ data collected from one subject that participated in the study. The decrease in total hemoglobin resembles the decrease in oxyhemoglobin compared with deoxyhemoglobin (Figure 3 and Figure 4). The recording of a subject who did not show any sign of change on NIRS during the hyperventilation test is revealed in Figure 5.

**Figure 6:**
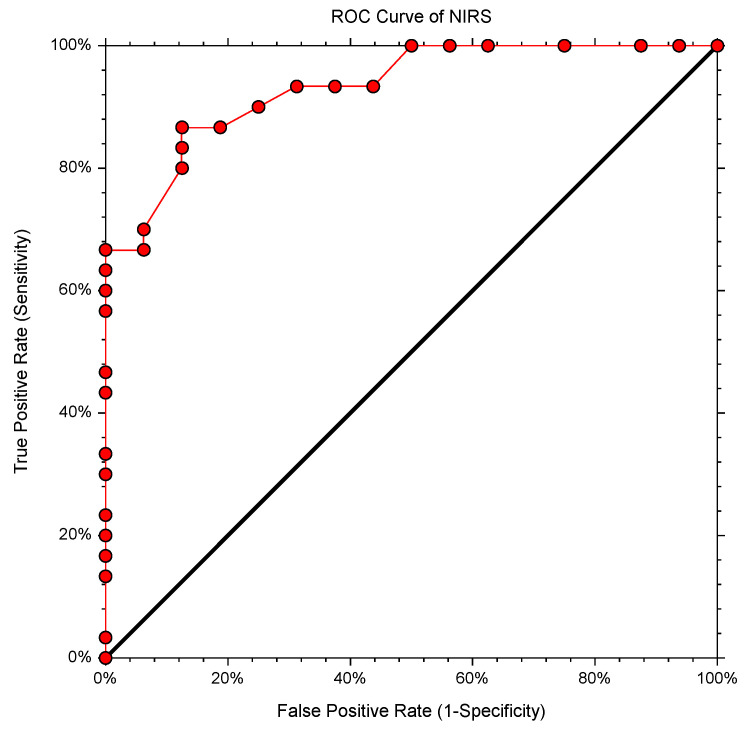
ROC curve analysis of ETCO_2_ to predict oximetry changes (NIRS).

We found that the cutoff value of ETCO_2_ decrease predicting changes in NIRS was ≥26%, with AUC-ROC (CI 95%) = 0.93 (0.8212–0.9753) (*p* < 0.0001), a sensitivity of 0.8667, a specificity of 0.875, a positive predictive value of 0.8678, a negative predictive value of 0.8739, and a Youden Index of 0.7417 (Figure 6).

The oximetry changes consisted of a reduction in oxyhemoglobin concentration, whilst the deoxyhemoglobin level remained almost constant. These subjects presented a reduction of 26% or greater from the baseline levels of ETCO_2_, along with oximetry changes, but an association between the percentage ETCO_2_ decrease and clinical signs was not mandatory. There were also subjects with a greater reduction in ETCO_2_ who did not report any signs.

## 4. Discussion

Our results showed that, during hyperventilation, reaching a certain prescribed respiratory rate (21.87 ± 3.58 breaths/min) higher than the initial baseline rate (10.45 ± 1.54 breath/min) was not necessarily followed by an equally significant rise in alveolar ventilation in a patient capable of sustaining a state of hypocapnia corresponding to changes in cerebral oximetry. ETCO_2_ levels allow accurately estimating the paCO_2_ values [20] and are conditioned by the tissue carbon dioxide production/alveolar ventilation ratio [21]. As such, ETCO_2_ recording is necessary because the respiratory rate cannot accurately estimate the alveolar ventilation (the amplitude of the respiratory movements is variable among subjects). 

Subjects maintained a constant ETCO_2_ at 2 min and 4 min, but most of them reported fatigue; hence, further respiratory effort could have led to a decrease in alveolar ventilation.

Assessment of hemodynamic cerebral changes during the hyperventilation test are essential in order to verify that the test is properly performed. There are many techniques used in humans that measure global or local cerebral blood flow, some invasive, based on brain pulsations recording through skull defects, and some less invasive or noninvasive, such as nitrous oxide dilution, transcranial Doppler ultrasound, magnetic resonance imaging, or NIRS [22,23,24]. In fact, NIRS is a modern technique that shows the tissue oxygenation index, the expression of the balance between oxygen delivered by the cerebral blood flow and local metabolic demands. It is known that hypocapnia secondary to hyperventilation induces cerebral arterial vasoconstriction as proven by imagistic studies [25]. Arterial vasoconstriction decreases the number of erythrocytes crossing the vessel, thereby also decreasing the amount of hemoglobin. This then determines an increase in the oxygen extraction ratio and a drop in oxyhemoglobin if the cerebral metabolism remains constant. 

We observed in our subjects a decrease in total hemoglobin (Figure 3 and Figure 4), and this decrease was similar to the decrease in oxyhemoglobin (Figure 3), while deoxyhemoglobin concentration remained constant (Figure 4). We preferred to monitor oxyhemoglobin and deoxyhemoglobin continuously during the test rather than to monitor the changes in total hemoglobin because the decrease in oxyhemoglobin is easier to observe compared to deoxyhemoglobin level, which remains constant. Thus, a decrease in total hemoglobin and oxyhemoglobin corresponds to arterial vasoconstriction. Similarly, in a study that focused on the effects of hyper/hypoxia and hyper/hypocapnia on cerebral tissue oxygenation, subjects that hyperventilated in order to reduce ETCO_2_ by 1.5 kPa below the baseline for 5 min had a reduced cerebral oxygenation index as a result of decreased cerebral blood flow and reduced cerebral blood volume [26]. Furthermore, a speech study revealed a decrease in ETCO2 by 4–10 mmHg during a 5 min recitation task, which was associated with oxyhemoglobin and total hemoglobin decrease [27]. The results were attributed to cerebral vasoconstriction secondary to hyperventilation, which prevailed over the neurovascular coupling mechanism associated with increased brain activity during the task.

Furthermore, we observed an association between the occurrence of cerebral arterial vasoconstriction, as established by NIRS cerebral oximetry, and the ETCO_2_ variation from baseline; specifically, oximetry changes indicating the occurrence of vasoconstriction were produced only in those subjects who also presented a reduction in ETCO_2_ level equal to or greater than 26% from baseline. Accordingly, we believe that ETCO_2_ monitoring is required, to have a more precise, quantifiable method that allows estimating the effectiveness and adequacy of the hyperventilation procedure as it is being conducted. 

Although ETCO_2_ measurements are used in emergency departments and intensive care units to early detect respiratory depression or the level of sedation [28], ETCO_2_ level is not always correlated with tissue oxygen saturation [29]. The NIRS-assisted oximetry readings raise the clinician’s awareness of recognizing the hyperventilation-related cerebral arterial vasoconstriction. This technique requires, however, trained personnel and involves supplementary costs compared to ETCO_2_ devices [30]. 

We must emphasize that there are systemic and brain variables that influence the measured cerebral saturation in oxygen. Confounding factors for brain oxygenation changes assessed by NIRS during hyperventilation test are the local neuronal activity and the neurovascular coupling reflex, vasomotricity of brain vessels in response to mean arterial pressure changes, muscle activity, and autonomic nervous system activation [31]. Recently, all these physiological systemic parameters have gained attention for fNIRS neuroimaging analysis, and a new method was developed, the systemic physiology augmented functional near-infrared spectroscopy (SPA-fNIRS), which allows a more comprehensive interpretation of the brain fNIRS signals [32]. 

Additionally, we observed that the presence of clinical symptoms described in many of the studies involving hyperventilation (e.g., dizziness, numbing of the extremities, or sensations of paresthesia) [33] was reported by seven of the subjects. All of them presented a reduction in ETCO_2_ equal to or greater than 26% coupled with changes in oximetry readings consistent with the occurrence of cerebral vasoconstriction. The absence of symptoms does not preclude effective hyperventilation. During anamnesis, it is important to inquire about relaxation techniques based on hyperventilation that the subject might practice, as such breathing relaxation methods are becoming increasingly popular [34]. In such patients, we did not notice any change in the cerebral oximetry despite the hyperventilation.

We believe that the cerebral activation test based on eliciting brain blood flow changes through hyperventilation is still an important method of evaluating cerebral reactivity, even in the face of other alternative minimally invasive methods [35,36]. However, for it to be adequately performed, the subject must be monitored with a cerebral oximeter and with a sensor capable of measuring ETCO_2_.

## 5. Conclusions

The vasoconstriction process in the brain’s circulatory system in response to hyperventilation is heterogeneous, with individual variations. The hyperventilation procedure utilized in clinical practice depends on many variables, and it should be backed by an ETCO_2_ decrease of at least 26% (AUC-ROC = 0.93) from the baseline. This is necessary because a respiratory rate that is double compared to baseline does not automatically translate into a significant rise in actual alveolar ventilation. Cerebral oximetry monitoring (NIRS) could also be included in the clinical protocol as a measure to validate the procedure. NIRS has the advantage of being a noninvasive technique providing useful real time information about vascular status in the investigating area. In some special situations, such as holotropic respiration, NIRS is requested because these kinds of patients seem to have other cutoff values. Regarding the length of the procedure, it should be between 2 min and 4 min. Further studies involving imaging are needed to confirm the ETCO_2_ cutoff for the hyperventilation test.

## Figures and Tables

**Table 1:** Values of respiratory rate and end-tidal CO_2_ during basal state and after 2 and 4 min of hyperventilation.

Parameter	Basal	Hyperventilation 2 Min	Hyperventilation 4 Min
Respiratory Rate	10.45 ± 1.54	21.87 ± 3.58	22.08 ± 3.58
ETCO_2_ (mmHg)	36.04 ± 4.49	25.89 ± 4.95	24.6 ± 5.85

## Data Availability

Not applicable.

## Appendix A

**Table A1:** The Recorded Parameters for All the Volunteers Enrolled in the Study: RR—Respiratory Rate, ETCO_2_—End-Tidal CO_2_.

Subject	Basal (RR)	RR at 2 Min	RR at 4 Min	Basal ETCO_2_	ETCO_2_ at 2 Min	ETCO_2_ at 4 Min
**II**	10	22	23	36	30	29
**VM**	12	24	22	42	32	34
**EC**	13	22	20	47	29	25
**CT**	12	24	19	38	27	32
**AA**	10	21	22	43	34	36
**PR**	10	21	19	38	28	24
**IA**	10	22	22	40	32	30
**BD**	12	20	24	40	31	31
**CD**	10	20	20	37	25	26
**MB**	12	24	20	34	28	30
**GM**	10	20	20	35	25	25
**NM**	10	20	20	34	22	18
**CZ**	10	20	22	30	18	17
**AG**	10	20	21	41	25	24
**AC**	10	20	22	33	20	19
**VM**	10	22	22	34	26	21
**AD**	10	20	21	28	20	19
**DF**	11	20	20	42	37	37
**CS**	10	20	19	38	32	31
**VLS**	10	20	20	37	30	29
**PB**	10	20	21	40	23	19
**MD**	12	23	23	40	26	23
**MM**	10	25	26	35	22	22
**NA**	9	27	25	30	19	19
**SD**	9	17	18	33	19	14
**PRI**	8	23	21	39	23	23
**DM**	10	23	26	32	30	26
**LC**	11	23	25	34	19	16
**PO**	10	25	24	40	22	21
**MA**	9	20	21	42	26	24
**PV**	10	19	26	40	28	19
**UA**	15	34	35	37	32	31
**BM**	10	20	23	39	26	25
**MP**	10	22	20	29	24	22
**AD**	14	33	30	34	25	26
**RB**	11	25	25	36	32	30
**TB**	10	24	25	34	26	24
**AN**	12	22	23	39	35	34
**ALI**	8	18	19	39	28	26
**AMIR**	11	20	20	31	24	23
**EF**	9	22	20	33	20	17
**CNI**	11	19	20	31	26	26
**DIL**	14	28	30	30	16	26
**CHT**	10	20	20	30	20	19
**CIC**	9	18	18	37	28	21
**RNS**	7	14	14	27	21	19

**Table A2:** NIRS Changes and the Amount of End-Tidal CO_2_ Variation for Each Subject.

Subject	NIRS Change	Delta CO_2_ (%)
**II**	Yes	20
**VM**	No	20
**EC**	Yes	39
**CT**	Yes	29
**AA**	No	21
**PR**	Yes	27
**IA**	Yes	20
**BD**	Yes	23
**CD**	No	33
**MB**	No	18
**GM**	Yes	29
**NM**	Yes	35
**CZ**	Yes	40
**AG**	Yes	40
**AC**	Yes	40
**VM**	Yes	24
**AD**	Yes	29
**DF**	No	12
**CS**	No	16
**VLS**	No	22
**PB**	Yes	53
**MD**	Yes	43
**MM**	Yes	37
**NA**	Yes	37
**SD**	Yes	57
**PR**	Yes	41
**DM**	No	19
**LC**	Yes	53
**PO**	Yes	48
**MA**	Yes	43
**PV**	Yes	53
**UA**	No	16
**BM**	Yes	36
**MP**	No	24
**AD**	Yes	26
**RB**	No	17
**TB**	No	29
**AN**	No	13
**ALI**	Yes	34
**AMR**	No	23
**EFL**	Yes	49
**CNIC**	No	17
**DIL**	Yes	47
**CHTAS**	Yes	37
**CCIOT**	No	25
**RNS**	Yes	30
